# Antibacterial activity and sensory properties of *Heracleum persicum* essential oil, nisin, and *Lactobacillus acidophilus* against *Listeria monocytogenes* in cheese

**DOI:** 10.14202/vetworld.2019.90-96

**Published:** 2019-01-17

**Authors:** A. Ehsani, A. Rezaeiyan, M. Hashemi, M. Aminzare, B. Jannat, A. Afshari

**Affiliations:** 1 Department of Food Science and Technology, Faculty of Nutrition, Tabriz University of Medical Sciences, Tabriz, Iran; 2 Department of Food Hygiene and Aquatics, Faculty of Veterinary Medicine, Urmia University, Urmia, Iran; 3 Department of Nutrition, Faculty of Medicine, Mashhad University of Medical Sciences, Mashhad, Iran; 4 Department of Food Safety and Hygiene, School of Public Health, Zanjan University of Medical Sciences, Zanjan, Iran; 5 Halal Research Center of IRI, FDA, Tehran, Iran

**Keywords:** Heracleum persicum, Lactobacillus acidophilus, Listeria monocytogenes, Nisin

## Abstract

**Aim:**

The aim of this study was to evaluate the antibacterial and chemical effect of *Heracleum persicum* essential oil (EO), nisin, *Lactobacillus acidophilus*, and their combination against *Listeria monocytogenes* both *in* *vitro* and in Iranian white cheese model.

**Materials and Methods:**

Chemical compositions of *H. persicum* EO were analyzed by gas chromatography–mass spectrometry. After production of Iranian white cheese, minimum inhibitory concentration (MIC) and minimum bactericidal concentration of EO and nisin and agar spot test of *L. acidophilus* against *L. monocytogenes* were evaluated.

**Results:**

Hexyl butanoate (25.98%), octyl isobutyrate (17.82%), methyl butyrate (14.37%), and pentyl cyclopropane (12.77%) were the main components of the EO. MIC of the EO against *L. monocytogenes* was 2.5 mg/mL. Combination of nisin (5.3 IU/mL) and *H. persicum* EO (2500 µg/mL) showed increasing effect against *L. monocytogenes* (fractional inhibitory concentration = 0.9), while a higher concentration of EO and nisin showed undesirable effect on the cheese flavor. Furthermore, a combination of 10^12^ CFU/g *L. acidophilus* with *H. persicum* EO at the concentration of 2.5 mg/mL (T12) showed acceptable sensorial and also antibacterial results in Iranian white cheese.

**Conclusion:**

Combination of *H. persicum* EO, *L. acidophilus*, and nisin can be recommended as natural preservatives and flavoring agents in cheese.

## Introduction

Today, people are more willing to consume foods with natural preservatives so-called green rather than with artificial preservatives [1]. Natural preservatives such as essential oils (EOs) are produced from some seasonings and herbs and have flavor enhancing and antimicrobial effects [2]. EOs are oily aromatic liquid(s), obtained from different parts of plants which are also called ethereal oils or volatile oils [3]. *Heracleum persicum* (Persian Hogweed or Golpar) is a plant which has been used in the preparation of food and medicine in Iran. The fruits and leaves of *H*. *persicum* are used to flavor pickles. It is also used as an antiseptic, analgesic, anti-flatulence, and digestive aid in Iranian traditional medicine [4]. It is noteworthy that, due to the impact of geographical climate on the components of *H. persicum*, there are various reports about chemical and, especially, anti-bacterial properties of some parts of this herb [5].

Nisin is a known antimicrobial agent and the only bacteriocins used as an additive to increase the shelf life of food in industries of >50 countries [6]. Combination of herbal EOs and extracts with nisin shows a synergistic effect on decreasing extracellular adenosine triphosphate in microorganisms [7]. On the other hand, the use of probiotics to enhance health and improve the digestive system has been proposed for decades. The probiotic bacteria (especially, different species of *Lactobacillus* and *Bifidobacterium*) are known to possess antimicrobial activity, and therefore, they can be used to control and prevent the growth of spoilage bacteria and food-borne pathogens instead of chemical and synthetic preservatives [8].

Hence, this study was conducted to determine the following: (1) The chemical composition and antimicrobial properties of the aerial parts of the *H. persicum* EO collected from the mountainous region in Northeastern of Iran, (2) *in* *vitro* antibacterial effect of *H. persicum* EO, nisin, and *Lactobacillus acidophilus* against *Listeria monocytogenes*,and (3) antimicrobial activity of *H. persicum* EO, *L. acidophilus*, and nisin against *L. monocytogenes* in Iranian white cheese.

## Materials and Methods

### Ethical approval

This study does not work on human or animal so, it does not need ethical approval.****

### Preparation and analysis of the EO

Aerial parts of the *H. persicum* plant were collected in full flowering stage in July 2014 from the mountainous regions: Sheykh Musa and Bandpei, Babol County, Mazandaran Province, Iran. They were kept away from sunlight at room temperature until completely dried before distillation [9]. Distillation operation was carried out by Clevenger Apparatus according to the method of water distillation. The extracted EOs were stored in dark glass container(s) in a refrigerator (4°C) for chemical and antimicrobial analysis [10].

Constituents of the *H. persicum* EO were determined by gas chromatography (GC) device (Agilent Technologies-7890A model) connected to mass spectrometry (MS) (Agilent Technologies-5975C model) with HP-5MS capillary column of 30 m (inner diameter of 0.25 mm and inner layer thickness of 0.25 μm). The initial temperature was set up at 60-280°C with a gradual increase of 4°C. The injection chamber temperature was 250°F and helium gas was used at a rate of 2 mL/min. Ionization energy parameters and temperature of ionization source were 70 eV and 270°C, respectively [11]. Separated chemicals were identified from their corresponding mass spectra using data available in the Wiley Library (Wiley-VCH 2001 data software, Weinheim, Germany).

### Bacterial preparation

The lyophilized cultures of *L. monocytogenes* PTCC1165 were transferred into brain heart infusion (BHI) broth (Merck, Germany) and incubated at 37°C for 18 h, with two consecutive transfers. Bacterial suspension was added to sterile cuvettes containing 5 mL of BHI broth, and the absorbance was determined at 600 nm using a spectrophotometer until achieving the concentration of 1.5×10^8^ CFU/mL (according to the 0.5 McFarland standard turbidity) and serially diluted to the desired concentration (1.5×10^6^ CFU/mL). The suspension was used for the inoculation of BHI agar or cheese samples.

### Nisin preparation

Amount of 10 g (10^6^ IU/g) of pure 2.5% nisin (balance sodium chloride) (Sigma–Aldrich Inc., United Kingdom), from *Lactococcus* lactis, was dissolved in hydrochloric acid 0.02 N to reach 10^5^ IU/mL. Then, it was sterilized using a 0.45 μm filter (Millex-HV Syringe Filter Unit) and was frozen at −20°C. The stock solution of nisin was thawed to 25°C and diluted in sterile water to obtain the desired concentrations for further analysis *in vitro* and in Iranian white cheese.

### Probiotic preparation

Lyophilized *L. acidophilus* (PTCC 1643) was prepared from Iranian Research Organization for Science and Technology. It was cultured on Man–Rogosa–Sharpe (MRS) broth under sterile condition and incubated anaerobically at 37°C for 24-48 h to achieve the desired bacterial count (10^9^ and 10^12^). The microbial cells were harvested and washed twice with sterile peptone water (0.1%) for inoculation into milk.

### *In vitro* antibacterial properties of *H. persicum* EO against *L. monocytogenes*

Antibacterial activity of *H. persicum* EO was evaluated against *L. monocytogenes*, using two methods: Microdilution and disk diffusion. For disk diffusion method, the amount of 100 μl of 1.5×10^6^ CFU/mL of bacterial suspension was cultured on BHI agar. Then, paper discs with a diameter of 6 mm were placed on each plate by sterile forceps. A concentration of 10 mg/mL of the EO was also prepared with methanol solvent. Then, 10 μl of the EO was spilled on each disk and incubated for 24 h at 37 °C. Finally, the diameter of inhibition zone around each disk was measured by a caliper. Antibiotics discs (ampicillin) were used as positive control [12].

Minimum inhibitory concentration (MIC) and minimum bactericidal concentration (MBC) values were also determined by microdilution method using 96-well microplates. The EO was prepared at the highest concentration (80 mg/mL) by dimethyl sulfoxide (10%) solvent, and the serial two-fold dilutions were made in the concentration range from 0.31 to 80 mg/mL in nutrient broth. Then, 160 μL of BHI broth, 20 μL of bacterial suspension at concentration of 1.5×10^6^ CFU/mL, and 20 μL of the EO (the final volume of each well: 200 μL, the final concentrations of the EO: 0.03-8 mg/mL, and the final bacterial level: ~1.5×10^5^ CFU/mL) were poured into the wells. Controls (without the addition of bacteria and without addition of the EO) were also prepared. Microplates were shacked at 2500 rpm for 30 s and kept at 37°C for 24 h. The lowest EO concentration that prevented bacterial growth (no visible bacterial growth) was considered as MIC value. To determine MBC value, 5 mL from wells with no visible growth in the MIC experiment was inoculated on BHI agar. The lowest concentration of the EO with bactericidal effects (lack of growth on the BHI agar) was considered as MBC value [10,12,13].

### *In vitro* antibacterial activity of *L. acidophilus* and nisin against *L. monocytogenes*

Agar spot test was used to evaluate the antimicrobial activity of *L. acidophilus* against mentioned pathogenic strain. *L. acidophilus* colonies that had been grown on MRS agar (Merck, Darmstadt, Germany) were inoculated on BHI agar containing 20 mM glucose and incubated at 37°C for 24 h under anaerobic condition (Anaerobic jar BBL, Cockeysville, Maryland, USA). After incubation, spots were transferred to the soft agar plates (BHI, 2% glycerol and 0.7% agar), which had been previously inoculated with 100 μl of 1.5×10^6^ CFU/mL of bacterial suspension. After 1 h at 37 °C in aerobic conditions, the plates were incubated for another 18-24 h at the aerobic condition, and the diameter of inhibition zone was measured by a caliper. Inhibition zones with a diameter of 1 mm or more were recorded as positive results [14].

Nisin with concentrations of 100, 200, 300, 400, 500, 800, 1000, 1500, and 2000 IU/mL in tryptic soy broth was prepared, and the microdilution assay was performed as previously described.

### *In vitro* antibacterial effect of combination use of the EO and nisin against *L. monocytogenes*

To assess the combined effects of nisin and the EO, fractional inhibitory concentration (FIC) value was used [15] as follows: Eight concentrations (five concentrations less than the MIC value, one equal with the MIC value, and two concentrations more than the MIC value) of *H. persicum* EO and nisin were prepared according to the microdilution method. Then, 140 μl BHI broth, 20 μl of bacterial suspension (final dose in each well: 1.5×10^5^), 20 mL of the EO concentrations, and 20 μl of nisin concentrations were poured into each well. The microplates were shaken for 30 s at 2500 rpm and incubated at 35°C for 24 h. Then, the MIC values of the EO and nisin were determined, and the FIC value was calculated using the following equation:

FIC of antibacterial substances = Combination MIC/MIC

FIC value (FIC index) ≤0.5 indicates a synergistic effect, between 0.5 and 1 indicates an increased effect, between 1 and 4 indicates no effect, and >4 indicates an antagonistic effect [16].

### Antimicrobial activity of the EO with *L. acidophilus* and nisin against *L. monocytogenes* in Iranian white cheese

#### Production of Iranian white cheese

Fresh cow milk was pasteurized at a temperature of 72±2°C for 15 s and was used for the production of Iranian white cheese. Desired dose of 10^3^ L *. monocytogenes* per milliliter of BHI broth was added into each sterile container containing 5 L of milk at a temperature of 35°C. 0.5% of starter culture (Chr. Hansen R 704), including *L. lactis* subsp. *cremoris* and *L. lactis* subsp. *diacetylactis*, was added to the milk [17]. Three concentrations of *L. acidophilus* (10^9^ and 10^12^) nisin (200, 400, and 800 IU/mL) and the EO (2.5, 5, and 10 mg/mL) were added to the milk samples according to the treatments in Table-1. Then, 0.02% of calcium chloride (CaCl_2_) in 20 mL of distilled water was added and dissolved in the milk at 40°C. To improve the efficiency of the rennet, the milk temperature was held at about 35°C during the clot formation. After 1 h, the clot was cut into 1-2 cm cubic pieces, and it was rehydrated under the pressure of 10 kg for 6 h, according to the Iranian white cheese preparation [17]. Then, rehydrated clots were kept in 22% brine (w/v) for 8 h. Clots were then transferred into 12% sterile salt water and kept at 14-16°C for 15 days. Microbial (bacterial enumeration) and chemical (determination of dry matter, acidity, and fat) analyses were performed on days: 0 (immediately after inoculation of bacteria in milk), 3, 7, 15 (at the end of cheese ripening period at 14-16°C), 30, 45, and 60.

**Table-1:** Treatments containing EO, nisin, and *L. acidophilus* in Iranian white cheese.

Treatments	Concentrations
T1-T3	EO concentration (2.5, 5, and 10 mg/mL) against *L. monocytogenes*
T4 and T5	*L. acidophilus* (10^9^ and 10^12^ CFU/mL) against *L. monocytogenes*
T6-T8	Nisin (200, 400, and 800 IU/mL) against *L. monocytogenes*
T9-T14	*L. acidophilus* (10^9^ and 10^12^ CFU/mL)+EO (2.5, 5, and 10 mg/mL) against *L. monocytogenes*
T15-T23	Nisin (200, 400, and 800 IU/mL)+EO (2.5, 5, and 10 mg/mL) against *L. monocytogenes*
T24	Control (without addition of nisin, EO, and *L. acidophilus*)

EO=Essential oil, *L. acidophilus=Lactobacillus acidophilus*, *L. monocytogenes=Listeria monocytogenes*

#### Bacterial enumeration

About 10 g of sample was placed in a sterile bag containing 90 mL of 0.1% sterile peptone water for homogenization using a Stomacher (Seward, London , UK). After serial dilutions preparation, Listeria selective agar (Merck) was used for surface colony counting at 37°C for 48 h [18]. It should be mentioned that the survivability of the probiotics was not evaluated in this study.

### Chemical analysis

Moisture and dry matter content analysis were performed at the end of the study by drying the samples at 102±2°C in an oven (Memmert, Schwabach, Germany), according to the following equation [19]:


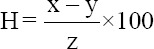


Where H: % Moisture; x: Weight of the container and sample before drying; y: Weight of the container and sample after drying; and z: Sample weight;

Moisture content = % Dry matter-100

Fat and protein contents were also determined by Gerber and Kjeldahl methods at the end of the study, respectively [18]. pH measurement was performed according to the method previously described by Sadler and Murphy [19] on days 0, 3, 7, 15, 30, 45, and 60.

### Sensory evaluation

Sensory characteristics of the treatments were evaluated by 9-point hedonic system by trained panelists who were selected from students and staff of the Department of Veterinary Medicine, Urmia University. Color, odor, texture, taste, and overall acceptability were evaluated on day 60 [20]. Treatments (500 g) containing different concentrations of the EO, probiotic, and nisin were prepared without inoculation of *L. monocytogenes*.

### Statistical analysis

All experiments were performed in triplicate. Statistical analysis was performed with SPSS software (version 18.0; IBM, Armonk, USA). Repeated measure and ANOVA analysis were used for comparing results between different groups. p<0.05 was considered as statistically significant.

## Results

### GC/MS analysis

Chemical compositions of *H. persicum* EO were determined by GC connected to MS and are shown in Table-2. The main constituents of the EO were butanoic acid, hexyl ester (hexyl butanoic) (25.98%), N-octyl 2-methyl butyrate (14.37%), pentyl cyclopropane (12.77%), and octyl isobutyrate (17.82%).

**Table-2:** Chemical constituents of *H. persicum* EO analyzed by GC/MS.

Compound	Retention time (min)	Amount (%)
Butanoic acid, butyl ester	5.618	0.7
Octanal	5.608	0.97
Isobutyl isovalerate	5.894	0.27
Hexyl acetate (acetic acid and hexyl ester)	6.159	2.88
Isopropylbenzene	6.381	1.47
Butyl 2-methylbutyrate	6.889	1.42
Butanoic acid, 3-methylbutyl ester	7.022	1.23
Gamma-terpinene	7.342	1.31
L-Linalool	8.636	0.3
β-Linalool	8.746	0.34
Hexyl propionate	8.813	0.35
Hexyl butanoate	11.941	25.98
Spiro [2.5] octane	12.03	2.49
Capraldehyde	12.14	0.28
Pentylcyclopropane	12.505	12.77
Octyl 2-methylbuyrate	13.246	14.37
Decyl isobutyrate	16.596	2.76
Vinyl cyclohexane	17.535	3
Octyl isobutyrate	18.11	17.82
2-(aminomethyl) butanoic acid	23.682	1.66
Angelicin	28.557	0.35
Octyl caprylate	28.911	0.24
Total		92.96

EO=Essential oil, *H. persicum*=*Heracleum persicum*, GC/MS=Gas chromatography/mass spectrometry

### In vitro antibacterial properties of *H. persicum* EO, nisin, and *L. acidophilus*

Results of *in vitro* antibacterial properties of *H. persicum* EO by disk diffusion method showed that growth inhibition diameters of EO (10 mg/mL) against *L. monocytogenes* were 6.7±0.15 mm. The inhibition zone for ampicillin, as control positive, was 28.2±0.25 and 17.4±0.45 mm for each of the treatments, respectively. Both MIC and MBC values of the EO were 2.5 mg/mL against *L. monocytogenes*. MIC and MBC of nisin against *L. monocytogenes* were 200 and 300 IU/mL by both methods. Growth inhibition zone of *L. acidophilus* was 13.2±0.15 mm by agar spot test.

Results of FIC values of *H. persicum* EO and nisin against tested bacteria are also shown in Table-3.

**Table-3:** FIC values of *H. persicum* EO (μg/mL) and nisin (IU/mL) against *L. monocytogenes.*

Microorganism	FICI	Nisin			Essential oil		
	
		FIC	MIC^c^	MIC^a^	FIC	MIC^c^	MIC^a^
*L. monocytogenes*	0.4+5.0	0.5	2.65	5.3	0.4	1000	2500

a Single antibacterial agent,

c Combination of antibacterial agents. FICI=Fractional inhibitory concentration index, *L. monocytogenes=Listeria monocytogenes, H. persicum=Heracleum persicum*, EO=Essential oil, MIC=Minimum inhibitory concentration

### Antibacterial effect of the EO in combination with *L. acidophilus* and nisin against *L. monocytogenes* in Iranian white cheese

Results of the effect of *H. persicum* EO, *L. acidophilus*, and nisin on the growth of *L. monocytogenes* during the storage of the Iranian white cheese are shown in Tables-4 and 5. Results indicated that, in the control group, bacterial counts reached to 8.10±0.04 log CFU/g at the end of the ripening period (day 60). All the treatments inhibited microbial growth when compared to the control (p *<*0.05).

**Table-4:** Antimicrobial results (mean±SD) of single treatments against *L. monocytogenes* viable cell count during 60 days of storage in Iranian white cheese.

Day/Treatments	Incubation time (days)							p-value*

	0 Mean±SD	3 Mean±SD	7 Mean±SD	15 Mean±SD	30 Mean±SD	45 Mean±SD	60 Mean±SD	
T1	3.4 (0.1)^a^	5.5 (0.09)^b^	6.6 (0.02)^c^	7.9 (0.009)^d^	7.4 (0.03)^e^	7.7 (0.02)^f^	8.1 (0.05)^g^	0.004
T2	3.2 (0.17)^a^	5.2 (0.11)^b^	6.4 (0.06)^c^	7.1 (0.03)^d^	7.3 (0.02)^e^	7.7 (0.02)^f^	7.9 (0.02)^g^	<0.001
T3	3.3 (0.05)^a^	4.9 (0.05)^b^	6.1 (0.01)^c^	6.6 (0.04)^d^	6.8 (0.08)^d^	7.5 (0.07)^e^	7.4 (0.06)^e^	<0.001
T4	3.1 (0.14)^a^	3.7 (0.04)^b^	4.4 (0.10)^c^	5.5 (0.04)^d^	5.6 (0.02)^e^	5.4 (0.04)^f^	5.3 (0.07)^f^	<0.001
T5	3.2 (0.06)^a^	3.8 (0.07)^b^	4.2 (0.17)^b^	4.8 (0.10)^b^	5.3 (0.04)^c^	5.1 (0.03)^d^	5 (0.06)^e^	<0.001
T6	3.1 (0.17)^a^	5.4 (0.15)^b^	6.5 (0.07)^c^	7.2 (0.01)^d^	7.5 (0.01)^e^	7.7 (0.05)^f^	7.9 (0.01)^g^	<0.001
T7	3.1 (0.15)^a^	5.4 (0.03)^b^	5.3 (0.08)^b^	5.1 (0.04)^b^	4.9 (0.07)^b^	4.7 (0.08)^c^	4.6 (0.04)^c^	<0.001
T8	3.1 (0.15)^a^	4.9 (0.04)^b^	5.1 (0.10)^b^	4.9 (0.04)^b^	4.8 (0.08)^b^	4.6 (0.05)^b^	4.4 (0.10)^c^	<0.001

* p value for difference with control. The same letters do not differ statistically by the Tukey test (p<0.05)

**Table-5:** Antimicrobial results of combination treatments against *L. monocytogenes* viable cell count during 60 days of storage in Iranian white cheese.

Day/Treatments	Incubation time (days)							p-value*

	0 Mean±SD	3 Mean±SD	7 Mean±SD	15 Mean±SD	30 Mean±SD	45 Mean±SD	60 Mean±SD	
T9	3.3 (0.06)^a^	3.8 (0.03)^b^	4.5 (0.07)^c^	5.2 (0.02)^d^	5.7 (0.01)^e^	5.5 (0.03)^f^	5.4 (0.03)^g^	<0.001
T10	3.1 (0.15)^a^	3.7 (0.12)^a^	4.6 (0.05)^b^	4.9 (0.05)^c^	5.6 (0.03)^d^	5.4 (0.03)^e^	5.3 (0.04)^f^	<0.001
T11	3.2 (0.17)^a^	3.6 (0.05)^a^	4.4 (0.10)^b^	4.9 (0.07)^c^	5.5 (0.04)^d^	5.2 (0.05)^e^	5.1 (0.01)^e^	<0.001
T12	3.3 (0.05)^a^	3.9 (0.05)^b^	4.5 (0.07)^c^	4.9 (0.04)^d^	5.5 (0.04)^e^	5.3 (0.04)^f^	5.2 (0.04)^f^	<0.001
T13	3.2 (0.07)^a^	3.7 (0.08)^b^	4.3 (0.10)^c^	4.9 (0.00)^d^	5.2 (0.02)^e^	5.1 (0.07)^e^	4.9 (0.06)^e^	<0.001
T14	3.1 (0.17)^a^	3.7 (0.15)^a^	4.2 (0.17)^a^	4.9 (0.07)^b^	5.2 (0.05)^b^	5 (0.06)^b^	4.8 (0.04)^c^	<0.001
T15	3.1 (0.15)^a^	5.3 (0.08)^b^	6.6 (0.02)^c^	7.1 (0.02)^d^	7.6 (0.11)^e^	7.7 (0.02)^e^	7.8 (0.00)^f^	<0.001
T16	3.1 (0.15)^a^	5.2 (0.04)^b^	6.5 (0.02)^c^	7.2 (0.01)^d^	7.5 (0.09)^f^	7.6 (0.06)^f^	7.7 (0.02)^g^	<0.001
T17	3.3 (0.05)^a^	5.1 (0.00)^b^	6.6 (0.02)^c^	7.3 (0.02)^d^	7.6 (0.08)^e^	7.7 (0.04)^e^	7.8 (0.01)^f^	0.01
T18	3.2 (0.05)^a^	5.2 (0.07)^b^	5.2 (0.03)^b^	5.1 (0.04)^c^	4.9 (0.07)^c^	4.8 (0.01)^c^	4.7 (0.04)^c^	<0.001
T19	3.2 (0.06)^a^	5.1 (0.19)^b^	5.2 (0.03)^b^	5.2 (0.02)^c^	4.9 (0.17)^c^	4.8 (0.01)^c^	4.7 (0.04)^c^	<0.001
T20	3.5 (0.06)^a^	5.3 (0.08)^b^	5.3 (0.02)^b^	5.1 (0.03)^c^	4.8 (0.04)^d^	4.6 (0.08)^d^	4.5 (0.06)^d^	<0.001
T21	3.4 (0.05)^a^	4.9 (0.04)^b^	5 (0.03)^b^	4.8 (0.08)^c^	4.6 (0.05)^c^	4.2 (0.17)^d^	4.1 (0.12)^d^	<0.001
T22	3.4 (0.03)^a^	4.8 (0.06)^b^	5.1 (0.05^c^	4.8 (0.06)^d^	4.7 (0.08)^d^	4.4 (0.10)^e^	4.1 (0.16)^f^	<0.001
T23	3.3 (0.10)^a^	4.9 (0.05)^b^	5 (0.03)^c^	4.7 (0.04)^d^	4.5 (0.07)^e^	4.2 (0.17)^e^	3.9 (0.02)^e^	<0.001
T24	3.2 (0.20)^a^	5.6 (0.05)^b^	6.5 (0.01)^c^	7.2 (0.01)^d^	7.6 (0.07)^e^	7.8 (0.01)^f^	8.1 (0.04)^g^	<0.001

* p value for difference with control. The same letters do not differ statistically by the Tukey test (p<0.05)

### Chemical analysis

Fat, protein and dry matter contents of the treatments were evaluated at the end of the storage period. In this respect, no significant difference was observed among different treatments, and all of them were in acceptable range.

### pH changes

The pH of the treatments was evaluated on days 0, 3, 7, 15, 30, and 60. The average pH of the milk used for the cheese production was equivalent to 6.75 on the 1^st^ day. On days 15 and 60 of storage, pH showed a decreasing trend, such that it reached to 4.98 and 4.74 in the control group. pH changes were significantly different in samples containing 400 and 800 IU/mL nisin and samples containing higher concentrations (10^12^) of probiotics compared to the control at the end of the storage period.

### Sensory analysis of Iranian white cheese in different treatments

Treatments containing 10^9^ L *. acidophilus* showed the best acceptable taste, while the worst result was observed in treatments with 200 IU/mL nisin and 10 mg/mL *H. persicum* EO and treatments containing 800 IU/mL nisin +10 mg/mL *H. persicum* EO (data not shown here).

## Discussion

Chemical substances such as secondary metabolites in plants, having bioactive and biochemical properties, are used in many different industries such as pharmaceutical, chemical, cosmetics, and food industry [21]. In addition to EOs and plant extracts, probiotics and bacteriocins are also natural preservatives that have attracted the attention of consumers and food manufacturers in recent decades.

In the present study, the entire aerial parts of *H. persicum* were analyzed, and four components of hexyl butanoate (25.98 %), octyl isobutyrate (17.73 %), n-octyl-2 methyl- butyrate (14.37%), and pentyl cyclopropane (12.77 %) were identified as the main constituents of the EO. Despite the differences with other studies regarding EO compositions [22-24], aliphatic esters were the main constituents of the *H. persicum* EOs. Climate, time of harvest, storage time, distillation method, and genetic differences are effective on the compositions and concentrations of plant EO [25]. Since EO application in foods is generally recognized as safe including *H. persicum* [26] and also based on the components detected in the GC analysis, this EO was chosen in this study.

To the best of our knowledge, there is only one study focusing on the antibacterial effect of the *H. persicum* EO, describing that *H. persicum* EO has some antimicrobial effects against *Escherichia coli* and *Campylobacter* *jejuni* by agar disc diffusion and microdilution assays [27]. The antimicrobial effect of *Heracleum thomsonii* against fungi, and Gram-positive and Gram-negative bacteria was also evaluated, and a strong antibacterial effect of *H. thomsonii* was reported, which could be due to the presence of significant amounts of different compositions such as “nerol” (87.9%) and “neryl acetate” (62.51%) [28].

MIC and MBC values of nisin for *L. monocytogenes* were 5.3 and 6.9 mg/mL, respectively.

A previous study showed that nisin had antimicrobial effect on *L. monocytogenes* in BHI broth; although antibacterial effect of nisin on *L. monocytogenes* is strain dependent [29]. Murdock *et al*. [30] reported MIC value of nisin against *L. monocytogenes* as 250 IU/mL. Different reported MIC values for nisin might be due to the different applied media; for example, in the Tryptose Agar medium, microorganisms show higher MIC value because the presence of high amounts of divalent cations causes more resistance to nisin [30]. Nisin creates holes in the membrane cytoplasm and disables the proton motive force which stops the absorption of amino acids and small metabolites [31].

Low stability of nisin at higher pH values has restricted its application in certain food products [32]. Therefore, nisin in combination with other natural antimicrobial is an innovative approach for its usage. According to the results of this study, combinational use of nisin with *H. persicum* EO showed stronger effects against *L. monocytogenes* than individual usage. There are no available published data about the application of nisin in combination with *H. persicum* EO against pathogenic bacteria, but there are reports about the use of nisin with other plant EOs and extracts. In a former study, the effects of organic extracts of garlic and nisin were evaluated on six strains of *L. monocytogenes* in a model broth medium, and a synergistic bactericidal effect was observed [33].

Increasing concentration of nisin (T21, T22, and T23) could significantly decrease the number of *L. monocytogenes* (p<0.05), but the use of nisin at higher doses might be high risk because of the development of resistant strains against bacteriocins [34], so it is necessary to use other natural preservatives such as EOs or probiotic bacteria to use lesser amounts of nisin.

The results of the effect of *L. acidophilus* against *L. monocytogenes* suggested a suitable inhibitory effect of this probiotic against tested pathogenic bacteria. In this regard, Strus *et al*. [35] reported anti-pathogenic impact of *Lactobacillus* strains against anaerobic pathogens of gastrointestinal tract. Niel *et al*. [36] reported the treatment of neonatal diarrhea with probiotic *Lactobacillus* and reported signs of improvement. Ogunbanw *et al*. [37] studied the antimicrobial activity of *L. plantarum* and *L. brevis* by well diffusion method, and these organisms exhibited preventive activities against *E. coli* NCTC10418 and *Enterococcus faecalis* EF1. Products of the probiotic bacteria such as acetic acid and lactic acid prevent the growth of pathogens by changing the pH value as well as inhibition of adhesion and invasion to the epithelial cell by increasing the production of intestinal mucins [38]. *L. acidophilus* also showed antimicrobial potency against *L. monocytogenes* through creating growth inhibition zones in an agar spot test.

Despite strong antimicrobial activity of EOs against pathogenic and food spoilage microorganisms, the practical use of EOs in food production industry has been limited, due to adverse flavor changes [39]. In this study, a combination of 10^12^ CFU/g *L. acidophilus* with *H. persicum* EO at the concentration of 2.5 mg/mL (T12) showed remarkable sensorial and also antibacterial results in Iranian white cheese.

Lactic acid bacteria, compared to other gram-positive bacteria, are highly resistant against EOs [40]. Hence, the effect of nisin and *L. acidophilus* on pH and cheese production process should be taken into consideration. All concentrations of the EO and lower levels of probiotic had no significant effect on pH value of the samples, while significant changes of pH value were observed in treatments containing 400 and 800 IU/mL of nisin and treatments containing higher levels of probiotic (data not shown here). The sensitivity of lactic acid bacteria against nisin is one of the logical reasons for changes in pH of treatments, containing higher concentrations of nisin (400 and 800 IU/mL). *L. acidophilus* can also produce bacteriocins, affecting starter bacteria and modifying the cheese ripening process. In a study by Kykkidou *et al*. [41], on Galotyri (Greek local soft cheese), lactic acid bacteria were sensitive to nisin, and along with the reduction of spoilage bacteria, the number of *Lactobacillus* and *Lactococcus* strains was also reduced in cheese.

## Conclusion

According to the obtained results, a combination of low concentration of *H. persicum* EO, *L. acidophilus*, and nisin can be suggested as flavoring agents and natural preservative(s) in the cheese production industry.

## Authors’ Contributions

AR and BJ carried out the experiment. AA wrote the manuscript. MA and MH supervised the project. AE conceived the original idea and supervised the project. All authors read and approved the final manuscript.
